# miR-TV: an interactive microRNA Target Viewer for microRNA and target gene expression interrogation for human cancer studies

**DOI:** 10.1093/database/baz148

**Published:** 2020-01-16

**Authors:** Chao-Yu Pan, Wen-Chang Lin

**Affiliations:** 1 Institute of Biomedical Informatics, National Yang-Ming University, Taipei 112, Taiwan; 2 Institute of Biomedical Sciences, Academia Sinica, Taipei 115, Taiwan

## Abstract

MicroRNAs (miRNAs) have been identified in many organisms, and they are essential for gene expression regulation in many critical cellular processes. The expression levels of these genes and miRNAs are closely associated with the progression of diseases such as cancers. Furthermore, survival analysis is a significant indicator for evaluating the criticality of these cellular processes in cancer progression. We established a web tool, miRNA Target Viewer (miR-TV), which integrates 5p-arm and 3p-arm miRNA expression profiles, mRNA target gene expression levels in healthy and cancer populations, and clinical data of cancer patients and their survival information. The developed miR-TV obtained miRNA-seq, mRNA-seq and clinical data from the Cancer Genome Atlas and potential miRNA target gene predictions from miRDB, targetScan and miRanda. The data presentation was implemented using the D3 javascript toolkit. The D3 toolkit is frequently used to provide an easy-to-use interactive interface. Our miR-TV provides a user-friendly and interactive interface, which can be beneficial for biomedical researchers to freely interrogate miRNA expression information and their potential target genes. We believe that such a data visualization bioinformatics tool is excellent for obtaining information from massive biological data.

Database URL: http://mirtv.ibms.sinica.edu.tw

## Introduction

Cancer is a major cause of death worldwide and a serious health and social concern. Globally, nearly one in six deaths is cancer-related. Thus, understanding molecular mechanisms underlying cancer cell growth, survival and metastasis is critical (1). Gene expression profiling is commonly used in cancer research to study molecular alterations in cancer cells. In addition to gene expression, regulation of gene expression is essential. MicroRNA (miRNA) is one such critical regulator of gene expression and cell differentiation (2). It functions in RNA silencing and posttranscriptional regulation of gene expression (3). The expression profiles of certain miRNAs and messenger RNAs (mRNAs) are closely related to cancer progression (4) and clinical outcomes (5). Integrated miRNA and mRNA target gene expression information would be useful for addressing dysregulated oncogenic networks in human cancers (6).

Several miRNA analysis tools have been developed, such as miRBase (7), miRDB (8), targetScan (9) and miRanda (10). They have useful miRNA target prediction algorithms and bioinformatics tools. However, none of them provide information on human diseases and an interactive visualization interface for biologists with limited computing skills. Regarding miRNA target gene expression, several gene expression tools for patient data analysis have been developed, such as Oncomine (11, 12), GENT (13), MERAV (14), cBioPortal (15), Human Protein Atlas (HPA) (16), Expression Atlas (17) and GEPIA (18). Oncomine, GENET and MERAV comprise microarray data sets only. Next-generation sequencing (NGS) data are used in cBioPortal, HPA and GEPIA. Specifically, cBioPortal consists of multiple genomic data types and displays genomic alterations between different types simultaneously. Although cBioPortal contains miRNA and mRNA data, it does not consider the relationship between miRNA and their mRNA targets. In addition, it uses old mRNA-seq data and mRNA-seq V2, rather than new versions of data. HPA focuses on tissue proteome. GEPIA is an information-rich tool, but miRNA expression, the gene expression of miRNAs targets, the clinical data of samples and survival information are not integrated by GEPIA or any of the other tools. In addition, these current databases provide predefined simple query options for users, which lack the flexibility required by most biologists. Furthermore, no current miRNA expression databases have clearly integrated 5p-arm and 3p-arm expression information.

Therefore, we attempted to combine target gene expression, 5p-arm and 3p-arm miRNA expression and clinical information in a more user-friendly web interface. Clinical outcomes and features are critical for analyzing cancer progression. Most biologists often prefer asking biological questions specific to their research field; furthermore, providing freedom for exploring miRNA and target gene expression information would be beneficial. In the era of big data, improving the user interface to allow users to explore and interrogate crucial knowledge within enormous data sets is essential (19). Thus, we attempted to develop a web-based interactive visualization tool that integrates these essential data sources to interrogate the relationship among miRNA, mRNA and clinical data. Therefore, miRNA Target Viewer (miR-TV) provides not only basic tools for simple statistical results, but also customization of tools for data interrogation. This enables users to modify the data displayed and interactively investigate the target mRNA gene expression and clinical information according to their criteria.

## Materials and Methods

### Data acquisition

The Cancer Genome Atlas (TCGA) program (20) data (version 18.0), which comprises miRNA, mRNA and clinical data, were downloaded from the Genomic Data Commons (GDC) data portal (https://portal.gdc.cancer.gov/) in August 2019. TCGA is a well-known large-scale cancer research program for studies on human cancer biology.

### Data source

Our miR-TV includes miRNA isoform and RNA-seq data (mRNA-Seq and miRNA-Seq) of 32 cancer types from TCGA. Glioblastoma multiforme lacks miRNA isoform data and FFPE Pilot Phase II lacks all RNA-seq data; therefore, both of them were excluded from miR-TV construction. The miRNA target data comprised miRDB (version 5.0), targetScan (version 7.2) and miRanda predicted results (August 2010 release). Only the intersected data from these prediction results were used in the miR-TV database.

### Processing of mRNA-Seq and miRNA-Seq data

We mapped the file name to a universally unique identifier (UUID) by using the application programming interface from the GDC and processed data using Perl. We performed statistical analysis on miRNA and mRNA data using the Student *t* test and Mann–Whitney *U* test. We obtained miRNA target prediction data from the results of miRDB (8), targetScan (9) and miRanda (10) algorithms. Only intersected data were included in our miR-TV database. Furthermore, we combined miRNA, mRNA and clinical data and designed an interactive visualization graphic user interface to enable biologists to examine crucial biological mechanisms in cancer cells.

### Webtool design

**Figure 1:**
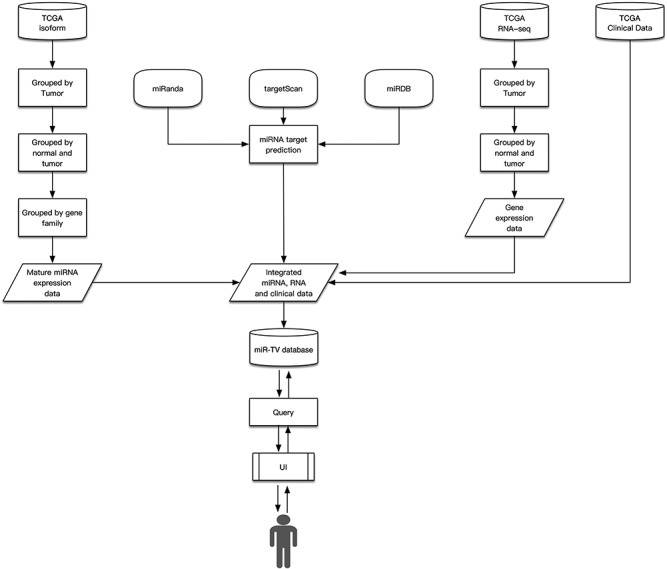
Design schema of miR-TV.

The miR-TV was developed using HTML5, PHP 7 (version 7.0.33) and javascript libraries such as the D3 javascript toolkit (version 3), JQuery (version 2.1.4) and Bootstrap (version 3.3.2). Gene expression analyses were performed using an R package. The database was established using MySQL (version 5.7.25). To accelerate data query, the expression and clinical data of all samples were divided into separate txt files according to their cancer type, miRNA or mRNA accession number and normal or tumor tissue classification. For example, the file isoform/BRCA/T/hsa_miR_100_MIMAT0000098.txt contained the miRNA MIMAT0000098 (miR-100) expression in breast cancer tumor tissue samples. The contents of the miRNA and mRNA interaction files were established in the same manner. When users query regarding a certain miRNA, miR-TV fetches the combination of expression and clinical data file directly, with no requirement for searching data in the SQL database, to generate quicker user responses. In addition, the total volume of all the combined expression and clinical data files was too large to be stored in the SQL database. The summary schema of the miR-TV design is presented in Figure 1.

### Statistical testing and data interrogation

We examined the expression of target genes by calculating the expression fold changes, log_2_ fold = (${\log}_2\frac{{\mathrm{tumor}}_{\mathrm{mean}}}{{\mathrm{normal}}_{\mathrm{mean}}}$); miR-TV performs the Student *t* test and Mann–Whitney *U* test on the expression of miRNAs and mRNA genes in normal and tumor samples and presents the *P*-value. Additionally, it provides dynamic Kaplan–Meier survival curves as a function. Users can select the data range freely; miR-TV provides the Kaplan–Meier survival curves and performs analysis to dynamically determine differences in the survival curves of the selected data compared with other data. This analysis is performed using the G-rho family of tests by Harrington and Fleming (21).

## Results

### Database contents and overview

Three main informative visualization features are displayed by miR-TV: the 5p-arm and 3p-arm miRNA expression levels of multiple cancer types, both summary and individual data, and real-time interactive interrogation data. Although current NGS data demonstrate the processing and utilization of both 5p- and 3p-arm data of mature miRNAs, little bioinformatic research has examined and presented 5p- and 3p-arm miRNA expression information (22). We developed miR-TV, the first bioinformatic database that simultaneously presents the comprehensive expression levels of 5p and 3p arms of any selected miRNA for all cancers in TCGA by using the newly established comprehensive 5p- and 3p-arm miRNA framework information from our laboratory (23, 24). Users can easily interrogate the specific miRNA expression levels of different tissues or cancers. In addition, users can compare the miRNA expression levels of either the 5p- or 3p-arm for any given miRNA.

### Data querying, searching, and browsing

Our miR-TV provides an intuitive, interactive, informative and simple web interface for miRNA and their mRNA target genes. We present a user interface that offers maximum information provision and simplicity. Specifically, miR-TV has three major functions: ‘searching and querying’, ‘miRNA and target gene data browsing’, and ‘dynamic visualization of clinical data’.

#### Searching and querying

Our miR-TV has a high-speed and easily implemented search function that provides candidate miRNAs from the miRbase list according to keywords input by the user.

#### Target gene data browsing and miRNA

**Figure 2:**
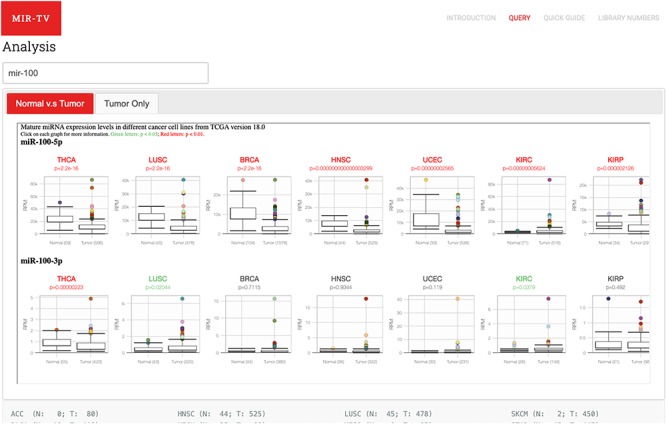
User query interface of miR-TV. First, users must enter the miRNA gene name in the query field. Once the miRNA is selected, both 5p- and 3p-arm expression box plot graphs of the selected miRNA in different cancer types are displayed in the main working window, and users can browse through their expression patterns by scrolling to the right side. Red labels indicate cancer tissues with a *P*-value < 0.01, and green labels indicate those with a *P*-value < 0.05. Several cancer types contain only tumor tissue samples (i.e. no normal counterparts); therefore, they are listed in the Tumor Only tab page.

**Figure 3:**
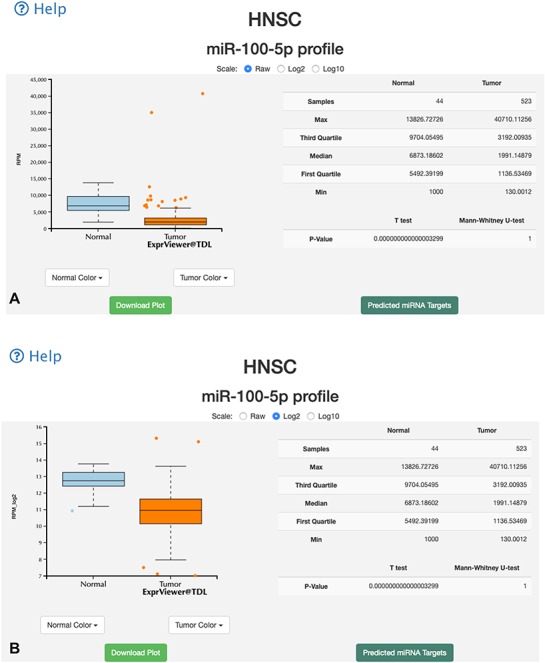

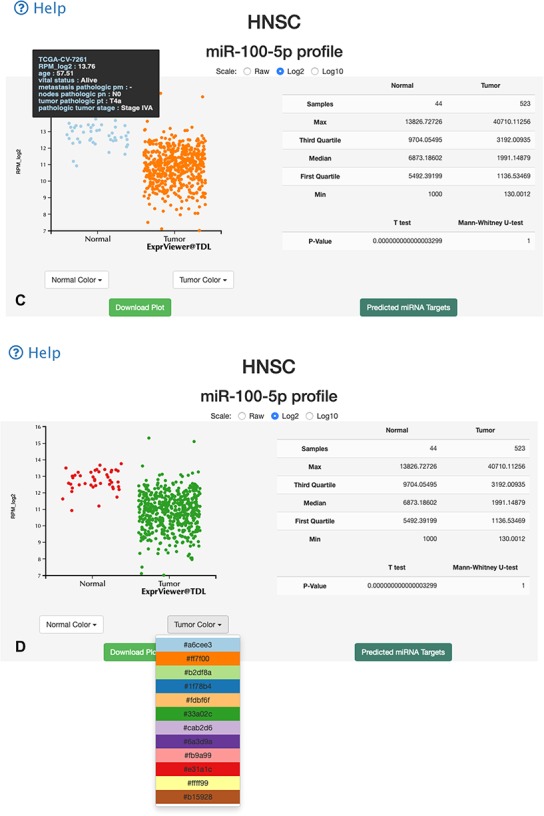
Overall-overview miRNA expression interface of miR-TV. (A) A new box plot graph window opens when the user clicks on a specific cancer type for interrogation. The default expression scale is RPM values. (B) Users can change the *y*-axis scale to a Log_2_ or Log_10_ scale by using the scale buttons at the top. (C) To obtain a better illustration of all patient samples, users can click on the box-shaped regions in either of the Normal or Cancer groups and the box plots will instantly transform into scatter plots. Double clicking the scatter plot smoothly reverts the scatter plot back to a box plot. Each dot represents one patient sample, and moving the mouse over each dot reveals TCGA sample ID, clinical information and miRNA expression value. (D) Two color change buttons are available below the graph plot area. Users can change the Normal group or Cancer group colors according to their preference.

Two data browsing interfaces are available. One is an overall-overview interface, wherein users select certain miRNA and miR-TV presents the expression level box plot of the selected miRNA in all TCGA cancer types (Figure 2). To identify differentially expressed miRNAs in different cancer types, red color labels indicate tissues with a *P*-value < 0.01 and green color labels indicate tissues with a *P*-value < 0.05. The number of tissue samples used for analysis of each cancer type is provided parenthetically for the normal and cancer tissues. Users can select a specific cancer type of interest. Subsequently, miR-TV provides a graphic box plot display panel and a data summary table, wherein detailed miRNA expression data is summarized, in the new overall-overview window (Figure 3A). Moreover, miR-TV simultaneously offers statistical descriptions and *P*-values. Our goal is to provide users with an easy method of freely interrogating the expression data and display style. In some cases, the gene expression values are too clustered to be seen clearly. We designed three scale buttons (Raw, Log_2_ and Log_10_) at the top of the graph. Users can manually change the scale of the expression value (Figure 3B). In addition, the box plot can be converted into a scatter plot by simply double clicking the mouse button. When the cursor hovers over any data point, miR-TV presents detailed information on that particular data point (Fig. 3C). Users can also change the display color scheme according to their preference. If users want to preserve the final custom plot following interrogation, they can save the jpg images on their device by simply clicking the download plot button (Figure 3D).

Several cancer types only contain tumor tissue samples; therefore, we have listed them in the Tumor Only tab page. These cancer types include ACC, DLBC, LAML, LGG, MESO, OV, SARC, TGCT, UCS and UVM. Users can view the expression of miRNAs in these cancer types but no differential expression information is displayed.

**Figure 4:**
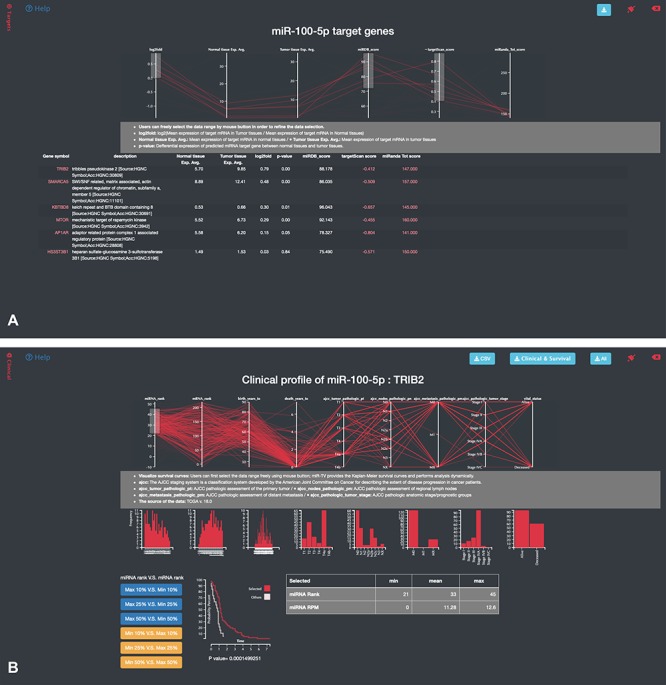
Specific-overview miRNA–target gene interface of miR-TV. (A) Users can enter the miRNA target gene interrogation interface by clicking either the green colored ‘Predicted mRNA Targets’ button or the @Targets black side menu bar. Multiple features of miRNA/mRNA expression and target prediction scores are displayed and can be easily filtered according to the user’s criteria by clicking and dragging the mouse to make a selection. Lines connect all information for any given target gene, and by moving the mouse over any given gene symbol, users can view the connected information, which is highlighted by a bold line. Additional prediction information is displayed by clicking the targetScan score and miRanda Tot score links; (B) by clicking on any target gene, users can enter the specific-overview interface for individual target gene interrogation. In this interface, users can further interrogate clinical information and miRNA/mRNA expression information by clicking the green colored ‘Clinical Information on Target mRNA’ button. Users can select different miRNA and mRNA expression groups to interrogate the expression relationship of miRNA–mRNA gene pairs. In addition, different clinical information features can be freely interrogated by clicking and dragging the mouse to make a selection. Preset buttons on the lower left panel can be used to examine the inverse relationship between the miRNA and target mRNA. The Kaplan–Meier survival curve plot and expression information is then displayed to dynamically reflect the user’s changes. The survival graph and detailed expression information can also be retrieved by using the download button on the top panel.

A specific-overview interface, the miRNA–target gene interface, is provided by miR-TV for the interrogation of miRNA target genes. By clicking the green-colored ‘Predicted miRNA Targets’ button or black side menu bar on the right side, the specific-overview interface is displayed (Figure 4A). The top panel displays the values of several vital features related to gene expressions and target predictions. Using the pointing devices, users can filter the data by simply selecting specific data ranges for each feature to interrogate candidate miRNA target genes. Such a selection box or boxes can be easily moved up or down and resized in order to filter the groups of data to be displayed (also displayed simultaneously in table on the web page), such as only interrogating target genes expressed at top levels. Fold changes in gene, normal tissue and tumor tissue miRNA expression levels and miRDB, targetScan and miRanda scores can be interrogated at the user’s discretion to further investigate potential target genes (Figure 4A). Additional prediction information (such as targeted regions and conservation and alignment information/scores) is displayed by clicking the targetScan score and miRanda Tot score links. By moving the mouse cursor over any given target gene in the target list table, the top graphic display panel dynamically displays the values of that particular gene.

#### Dynamic visualization of clinical data

Users can click on the any target gene to open a new protein mRNA gene-specific interrogation interface. In this target gene interrogation interface, gene expression information is displayed on a similar box plot interface. Box plots present the expression levels of this particular target gene in the normal and tumor samples in TCGA. Users can easily obtain more information regarding the distribution of target gene expression in the normal and tumor tissues. The box plot can be converted into a scatter plot by simply double clicking the box region. To obtain more information regarding the clinical data, the green colored button (labeled by ‘Clinical Information on Target mRNA’) or the black Clinical side menu bar on the right side can be clicked to display a specific-overview interface. Users can examine the miRNA and mRNA expression ranking, age at diagnosis, survival time following diagnosis, ajcc T score (ajcc_tumor_pathologic_pt), ajcc N score (ajcc_nodes_pathologic_pn), ajcc M score (ajcc_metastasis_pathologic_pm), tumor stage (ajcc_pathologica_tumor_stage) and vital status for advanced studies (Figure 4B). After selecting the features of interests, miR-TV instantly provides a dynamic display of a histogram or bar chart for each feature. More than one feature can be studied. Users can freely interrogate a combination of relevant features. Six preset buttons are available for users to initially examine the inverse expression relationship between miRNA and mRNA, such as by evaluating samples with the top 10% for miRNA expression levels that are also found to have the target gene expression in the bottom 10% for mRNA target gene expression levels. Furthermore, fine adjustment of the miRNA or mRNA ranges can be performed by using the mouse to click and drag any of the features in the top panel. In addition, miR-TV can further display the survival curves of patients with the selected ranges in any combined feature orders. Finally, users can download the clinical information/survival plots and raw data of expression for further analysis with the convenient download buttons on the top right panel (Figure 4B).

**Figure 5:**
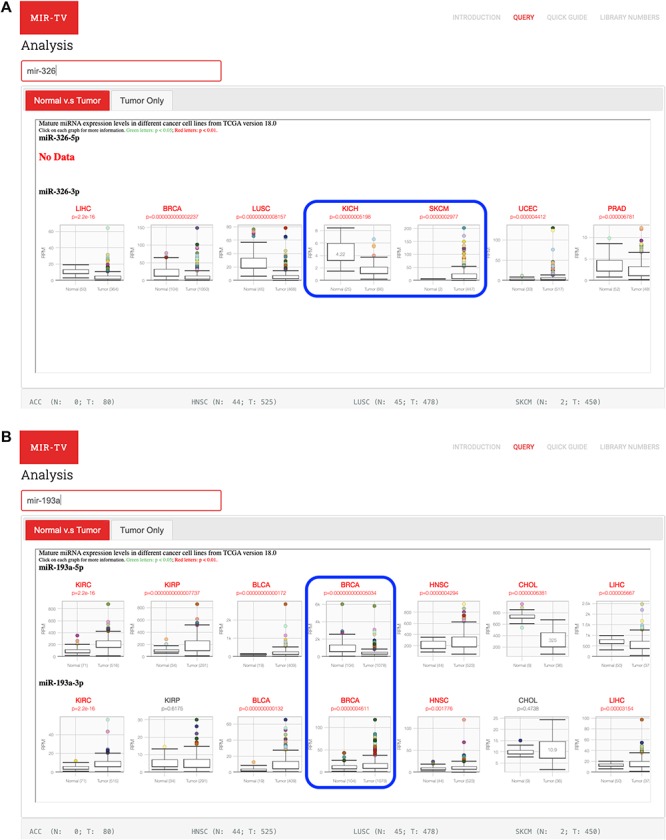
Examples of miRNAs and their targets using miR-TV interfaces. (A) Opposite miR-326 expression profiles in kidney cancers (KICH) and skin cancers (SKCM); (B) Differential miR-193a 5p- and 3p-arms expression profiles in breast cancer samples.

Potential tissue-specific expression patterns of selected miRNA can be presented by miR-TV. For instance, miR-326 expression is higher in normal tissues than in tumor tissues in kidney cancer, whereas it is higher in tumor tissues than in normal tissues in skin cancer (25) (Figure 5A). In the predicted target gene interrogation interface of the miR-326 3p-arm, SMTNL2 was found to have approximately 20-fold downregulation and satisfactory scores for miRDB and targetScan, which are known to be downregulated in kidney cancer (26). Other interesting observations can be made regarding miRNA arm-switching (27) by using miR-TV tools, namely the different miRNA 5p- and 3p-arms expression levels of specific miRNAs in different tissue types. For example, the expression levels of the 5p-arm and 3p-arm of miR-193a are different in breast cancer (Figure 5B). Similar findings were reported in another study (28).

## Conclusions and future perspectives

As crucial therapeutic molecules (29), miRNAs play critical roles in human cancers (30). Our miR-TV web database integrates mRNA target gene expression, arm-specific miRNA expression and clinical information from TCGA data sets, thus providing comprehensive insight into miRNA–mRNA regulation. Data visualization using interactive web tools can greatly improve data comprehension and exploration for general biologists.

We utilized the D3 javascript based parallel coordinate interactive display to provide an easy-view interactive display to connect various features and data points in one window. We integrated mRNA gene information, mRNA expression values, differential expression information and target gene prediction information for target genes. By moving the mouse over any given gene symbol, users can view relevant information of that specific gene, which is highlighted by a brighter bold line. The clinical data display integrates the associated clinical information of patient samples for particular miRNA–mRNA target gene pairs. The lines connect all information for any selected clinical sample. In this parallel coordinate interactive display, users can easily obtain information regarding the miRNA expression, mRNA target gene expression and relevant clinical information of each clinical sample. Users can select different miRNA and mRNA expression groups to interrogate the expression relationship of miRNA–mRNA gene pairs. Additionally, users can select different clinical information groups, such as high tumor stage and metastasis status groups, to further examine the difference in expression of miRNA–mRNA pairs. In miR-TV, we implemented an interactive web tool using D3 javascript toolkit, which allows biomedical researchers to interrogatively obtain miRNA information. In the future, a greater volume and variety of biological data can be integrated into miR-TV, such as gene ontology, pathway, proteomic, drug and therapy response information. Our miR-TV is a new type of web database that focuses on biological data presentation with the aim of reorganizing available biological data and presenting them in an intuitive, interactive, informative and simple manner.
